# Ras Signaling Inhibitors Attenuate Disease in Adjuvant-Induced Arthritis *via* Targeting Pathogenic Antigen-Specific Th17-Type Cells

**DOI:** 10.3389/fimmu.2017.00799

**Published:** 2017-07-07

**Authors:** Morad Zayoud, Victoria Marcu-Malina, Einav Vax, Jasmine Jacob-Hirsch, Galit Elad-Sfadia, Iris Barshack, Yoel Kloog, Itamar Goldstein

**Affiliations:** ^1^Sheba Cancer Research Center, Chaim Sheba Academic Medical Center, Ramat Gan, Israel; ^2^Rheumatology Unit, Chaim Sheba Academic Medical Center, Ramat Gan, Israel; ^3^Sackler School of Medicine, Tel-Aviv University, Tel-Aviv, Israel; ^4^Department of Neurobiology, The George S. Wise Faculty of Life Sciences & Sagol School of Neuroscience, Tel-Aviv University, Tel-Aviv, Israel; ^5^Institute of Pathology, Chaim Sheba Academic Medical Center, Ramat Gan, Israel

**Keywords:** Ras GTPases, rheumatoid arthritis, farnesylthiosalicylic acid, adjuvant induced arthritis, T-helper cells

## Abstract

The Ras family of GTPases plays an important role in signaling nodes downstream to T cell receptor and CD28 activation, potentially lowering the threshold for T-cell receptor activation by autoantigens. Somatic mutation in *NRAS* or *KRAS* may cause a rare autoimmune disorder coupled with abnormal expansion of lymphocytes. T cells from rheumatoid arthritis (RA) patients show excessive activation of Ras/MEK/ERK pathway. The small molecule farnesylthiosalicylic acid (FTS) interferes with the interaction between Ras GTPases and their prenyl-binding chaperones to inhibit proper plasma membrane localization. In the present study, we tested the therapeutic and immunomodulatory effects of FTS and its derivative 5-fluoro-FTS (F-FTS) in the rat adjuvant-induced arthritis model (AIA). We show that AIA severity was significantly reduced by oral FTS and F-FTS treatment compared to vehicle control treatment. FTS was as effective as the mainstay anti-rheumatic drug methotrexate, and combining the two drugs significantly increased efficacy compared to each drug alone. We also discovered that FTS therapy inhibited both the CFA-driven *in vivo* induction of Th17 and IL-17/IFN-γ producing “double positive” as well as the upregulation of serum levels of the Th17-associated cytokines IL-17A and IL-22. By gene microarray analysis of effector CD4^+^ T cells from CFA-immunized rats, re-stimulated *in vitro* with the mycobacterium tuberculosis heat-shock protein 65 (Bhsp65), we determined that FTS abrogated the Bhsp65-induced transcription of a large list of genes (e.g., Il17a/f, Il22, Ifng, Csf2, Lta, and Il1a). The functional enrichment bioinformatics analysis showed significant overlap with predefined gene sets related to inflammation, immune system processes and autoimmunity. In conclusion, FTS and F-FTS display broad immunomodulatory effects in AIA with inhibition of the Th17-type response to a dominant arthritogenic antigen. Hence, targeting Ras signal-transduction cascade is a potential novel therapeutic approach for RA.

## Introduction

Rheumatoid arthritis (RA) is a chronic systemic inflammatory autoimmune disorder that principally affects synovial joints (1). Th17 cells have been postulated to play a key role in the pathogenesis of several autoimmune diseases (2) and in animal models of human autoimmune diseases including autoimmune colitis (3), experimental autoimmune encephalomyelitis (4), collagen-induced arthritis (5), and rat adjuvant-induced arthritis (6).

Ras-GTPases are molecular switches that regulate key cellular processes, such as proliferation, differentiation, apoptosis, and motility. In T cells, Ras-family GTPases (e.g., K/N-Ras) are crucial for proper T-cell receptor (TCR)-dependent activation following antigen recognition. Defective activation of Ras/Raf/MEK/ERK1/2 cascade has been associated with T cell anergy, and accordingly increased expression of active Ras was shown to reverse anergy and to restore IL-2 production (7–9). Moreover, T cells from patients with RA have been found to express significantly higher levels of K-Ras and its downstream effector B-Raf that mediate the increased levels of phospho-ERK1/2 observed in RA patients’ T cells (10, 11). Importantly, over expression of K-Ras in CD4^+^ T cells from healthy donors enabled the induction of autoreactive T cells that reacted citrullinated vimentin-derived peptides, postulated to be a pathogenic autoantigen in RA. Thus, hyperactivity of the Ras signal transduction cascade has been postulated to increase TCR-sensitivity to low-affinity antigens, including many clinically relevant autoantigens (10, 11). Interestingly, previous reports suggest that inner plasma membrane (PM)-anchored Ras proteins can transfer from antigen-presenting B cells to T cells both through the immunological synapse and through actin supported long-range PM extensions termed tunneling nanotubes. These findings further support the importance of Ras GTPases in immunity, by showing that active Ras-GTPase signals can spread between immune cells (12, 13) and even from cancer cells expressing oncogenic Ras to T cells (14).

Activating mutations in the proto-oncogenes *KRAS* and *NRAS* are frequent in human cancers (15, 16). This has led to ongoing efforts to develop drugs that target Ras signaling (16–20). To be active, Ras GTPases have to associate with membranes, and hence they require several posttranslational modifications in their carboxy-terminal domain, such as the addition of the hydrophobic farnesyl isoprenoid molecule to Cysteine 186 that is conserved in all Ras family members (16, 21–23). Based on an innovative concept, Kloog and colleagues (24, 25) discovered a potent non-toxic inhibitor of active (GTP-bound) Ras proteins, the small molecule farnesylthiosalicylic acid (FTS/Salirasib).

In recent years, it has been discovered that following posttranscriptional processing Ras proteins interact with prenyl-binding chaperones (26–29). These chaperones with prenyl-binding hydrophobic pockets are vital for proper PM localization and effective downstream Ras signaling (30). In agreement with this concept, it was found that FTS, by competing for Ras-chaperon interactions, effectively dislodges the oncogenic Ras proteins from the PM and inhibits Ras mediated oncogenesis (31–33).

The central role of Ras signaling in T cells strongly suggests that targeting Ras might be an effective therapeutic approach for this disease. Over the past decade the effects of FTS and related analogs have been extensively studied in multiple pre-clinical animal models of autoimmune. For example, FTS can attenuate disease manifestations in experimental autoimmune encephalomyelitis (34, 35), Type 1 diabetes in NOD mice (36), experimental colitis (37), and other autoimmune diseases such as systemic lupus erythematosus (38). Preliminary studies by Aizman et al. (39) in the adjuvant-induced arthritis (AIA) model in rats suggest that prophylactic treatment with FTS may attenuate the clinical score of the disease; however, the biology behind the effect of FTS was not comprehensively elucidated. AIA is an experimental animal model of polyarthritis, which can be induced in inbred Lewis rats by immunization with Complete Freund’s adjuvant containing *Mycobacterium tuberculosis* (Mtb). Importantly, mycobacterial heat-shock protein 65 (Bhsp65) reactive T cells have been implicated in the pathogenesis of AIA. The AIA model and human RA have many overlapping characteristics, such as genetic susceptibility, T cell dependence, and pathogenic contribution of synovial CD4^+^ cells. Therefore, this model has been extensively employed for preclinical testing of numerous anti-arthritic agents, including biologics used for latest therapy in RA (40, 41). As previous studies imply that the main mechanism of action of FTS is down modulation of the T cell response (36), and the major role of T cells in AIA pathogenesis (42), we chose this pre-clinical model to assess the therapeutic potential of FTS in human RA.

Here, we provide a comprehensive insight into the molecular mechanisms that mediate the therapeutic action of small molecule Ras-inhibitors in AIA. Moreover, we determined that prophylactic treatment with FTS as an add-on to methotrexate (MTX) inhibits almost completely the development of AIA by all clinical and immunological/molecular outcome measures.

## Materials and Methods

### Animals

Lewis rats obtained from Harlan Biotech (Rehovot, Israel). All rats were subjected to regular health status controls. Male rat, 8 weeks of age were used for experiments. All animal experiments were conducted in accordance with relevant laws of the state of Israel and guidelines of the Tel Aviv University and approved by the Institutional Animal Care and Use Committee (Approval # L-14-018).

### Arthritis Induction and Drug Administration

To induce AIA, the rats have been injected intradermal (i.d.) at the base of the tail with 100 µl CFA produced by suspending heat-killed *M. tuberculosis* (Difco) in mineral oil at 10 mg/ml. For drug administration, on day +1 after AIA induction, the rats were randomly divided into four groups. Rats in the first group were treated daily (starting on day 1 after AIA induction) with oral (intragastric) FTS (100 mg/kg). Rats in the second group (control) received oral vehicle [0.5% carboxy methyl cellulose (CMC)] daily. Rats in the third group were treated with i.p MTX (0.5 mg/kg) on days 3, 10, and 17 after AIA induction. Rats in the fourth group (FTS + MTX) were treated daily (starting on day 1 after AIA induction) with oral (intragastric) FTS (100 mg/kg) as well with i.p MTX (0.5 mg/kg) on days 3, 10, and 17 after AIA induction. 20 days after the experiment has been terminated. As negative control, we used naïve healthy rats without any treatment. For determining the clinical scores, each paw is scored on a scale of 0–4 for the degree of swelling, erythema, and deformity of the joints with a maximum total score of 16 (43).

### Lymphocyte Isolation, Intracellular Cytokine Staining, and Flow Cytometric Analysis

Single-cell suspensions of splenocytes and inguinal lymph nodes (LN) were prepared by mechanical disaggregation followed by lysis of red blood cells with a commercial ammonium chloride buffer (Gibco, Thermo Fisher Scientific, Inc.). T cells were stained as indicated with the following anti-rat CDs monoclonal antibodies (mAbs): CD3-PE, CD4-FITC, CD8-PE-Cy7, and CD25-PE (all from eBioscience). For intracellular cytokine staining, freshly isolated lymphocytes were activated for 5 h *ex vivo* in RPMI medium with 1× Cell Stimulation Cocktail in the presence of a commercial Protein Transport Inhibitor Cocktail according to the manufacturer’s protocol (eBioscience). Thereafter, cells were surface-stained with anti-CD4-FITC, washed, fixed, and permeabilized using BD Cytofix/Cytoperm™ kit, per manufacturer’s instructions (BD Biosciences). Intracellular staining was performed with anti-rat IL-17A-PE and IFN-γ-APC (both mAbs from eBioscience) and the BD Perm/Wash™ Buffer. For Treg cells staining, the cells were surface stained for CD4 and CD25, washed, fixed, and permeabilized using the Foxp3 Staining Buffer Set, and then immunostained with anti-mouse/rat Foxp3-APC mAbs (all reagents from eBioscience). Samples were acquired using a FACSAria flow cytometer (BD Bioscience) and further analyzed using the FlowJo v10 software (TreeStar, Inc.).

### Measurement of Cytokines and CRP in Serum and Cell Culture Supernatant by ELISA

The serum levels of the inflammatory mediator CRP were determined by the rat specific CRP Elisa Kit (R&D Systems, Inc.). The levels of cytokines IL-17A and IL-22 from the collected rats sera and in cell culture supernatant were determined by rat specific IL-17A (homodimer) ELISA Ready-SET-Go kit (eBioscience) and by the rat IL-22 ELISA Kit (R&D Systems, Inc.), respectively.

### Processing and Evaluation of Joint Histology

At study termination, the tibiotarsal joint was transected at the level of the medial and lateral malleolus for Histopathological Assessment. Ankle joints were then collected into 4% paraformaldehyde, for at least 24 h, and then placed in a decalcifier solution with 10% hydrochloric acid. When decalcification was completed, the ankle joint was transected in the longitudinal plane and joints were processed for paraffin embedding, sectioned, and stained with hematoxylin and eosin. Tissue section slides of arthritic ankles were examined by an experienced pathologist, blinded to the animal treatment protocol, and scored for inflammation and bone resorption on a scale of 0–5, as previously described (44).

### Antigen-Specific T Cell Activation and Cell Cultures

Freshly isolated splenocytes were plated in 24 well plates at 2 × 10^6^ and re-challenged *in vitro* with Bhsp65 (5 µg/ml) or control vehicle, and cultured for additional 72 h in complete RPMI medium supplemented with 10% FCS. At culture termination, we first collected the supernatants for relevant analyses and then harvested the cells and isolated the CD4^+^ T cell population using Rat CD4 MicroBeads and the MACS cell separation platform (Miltenyi Biotec, Germany), according to the manufacturer’s instructions.

### GeneChip Microarray Analysis

The purified CD4^+^ T cell was lysed with the TRIzol^®^ Reagent (Invitrogen, Carlsbad, CA, USA), and total RNA was purified using the Direct-zol™ RNA Kit (Zymo Research Corporation, Irvine, CA, USA) for subsequent downstream analysis. Gene expression was determined using the GeneChip^®^ Rat Gene 2.0 ST Array System (Affymetrix, Inc.), according to the manufacturer’ instructions. The 2100 Bioanalyzer (Agilent Technologies) was used to determine RNA quality, and biotinylated target DNA was prepared from each suitable RNA sample according to the manufacturer’s instructions. Gene level RMA sketch algoritm was used for crude data generation (Affymetrix Expression Console and Partek Genomics Suite 6.2). Genes were analyzed using unsupervised hierarchical cluster analysis (Spotfire DecisionSite for Functional Genomics; Somerville, MA, USA) to get a first assessment of the data, and filtered according to fold change calculations. For further functional bioinformatics analysis to discover molecular processes and biological pathways enriched in the experimental dataset, we used the Gene Set Enrichment Analysis (GSEA) software available online (http://software.broadinstitute.org/gsea/msigdb/annotate.jsp). The primary microarrays data from this research have been deposited in the NCBI Gene Expression Omnibus data repository under accession number GSE100280.

### Reverse Transcription and Real-time qPCR

Total RNA was extracted from CD4^+^ cells using TRIzol^®^ Reagent (Invitrogen, Thermo Fisher Scientific, Inc.), and total RNA was purified using Direct-zol™ RNA Kits (Zymo Research Corporation), followed by treatment with 1 U of RNase-free DNase (Roche). Reverse transcription reactions were performed on 1 μg total RNA using the High Capacity cDNA Reverse Transcription Kit (Applied Biosystems, Thermo Fisher Scientific, Inc.), according to the manufacturer’s instructions. The quantitative PCR (qPCR) analyses for the relative mRNA expression of Ccl20, Il22, Il17A, Il17F, and Csf2 (normalized to Actb) were performed with premade QuantiTect bioinformatically validated primer sets (from Qiagen) and the SYBR Green Real-Time PCR Kit (Applied Biosystems) on an ABI Prism 7900 SDS Instrument (Applied Biosystems), as recommended by the manufacturers, in reactions containing 50–100 ng cDNA. All reactions were done in triplicates and relative mRNA quantities (RQ) were determined using the 2^−ΔΔCt^ method.

### Statistical Analysis

Statistically significant differences between group means were determined either by the one-way ANOVA test with Bonferroni’s *post hoc* multiple comparison test or the Student’s *t*-test, as appropriate, using the Prism win V.5.02 software (GraphPad Software, Inc.).

## Results

### FTS Treatment Attenuates Disease Severity in AIA

**Figure 1:**
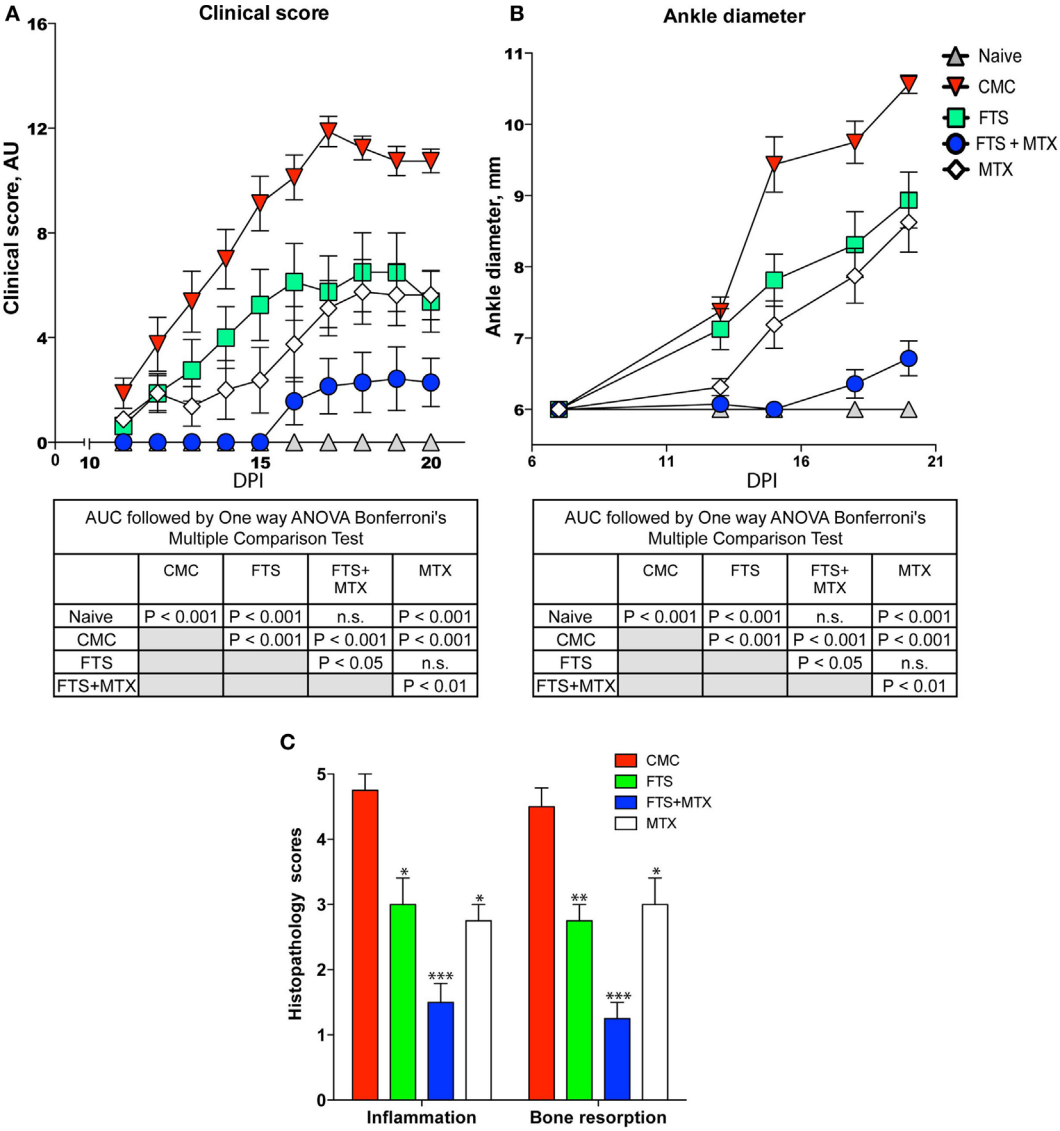
Farnesylthiosalicylic acid (FTS) treatment reduces the severity of adjuvant-induced arthritis. Rats were immunized with CFA and then graded regularly for signs of arthritis by a clinical disease severity score **(A)**, and by caliper measurements of ankle joint diameter **(B)**. Rats in the experimental arms were treated starting day +1 with either oral FTS (100 mg/kg), 0.5% carboxymethyl cellulose (CMC) vehicle solution, weekly i.p injection of MTX (0.5 mg/kg), or FTS combined with MTX. Statistical analysis of the AUC of the effect of the various treatments on the clinical score **(A)** and ankle diameter **(B)** was done by one way ANOVA with *post hoc* Bonferroni’s multiple comparison test. The results of the analysis are summarized in the table inset. One representative of three independent experiments is depicted (*P ≤ 0.05, **P ≤ 0.01, and ***P ≤ 0.001). **(C)** Tibiotarsal (ankle) joint tissue sections were stained with H&E for histopathological assessment, and scored on a scale of 0–5 for inflammation and bone resorption. The bar graph represents the quantification and statistical analysis of histopathology of ankles of arthritic rats in the various treatment arms. Shown bars from >3 experiments performed with *n* = 8 rats in each treatment arm.

To determine whether treatment with FTS suppresses the clinical signs of AIA, we started daily treatment of rats from day +1 after immunization in the experimental arm with oral FTS (100 mg/kg). Control rats received oral solution of 0.5% CMC (vehicle). As a “positive control,” we treated a group of Lewis rats with weekly *i.p* injection of the anti-rheumatic drug MTX (0.5 mg/kg). AIA progression and severity was scored on a clinical index of 0–16 (0–4 scale for each paw). In parallel, we also assessed arthritis severity and progression by successive caliper measurements of ankle joint diameter. We found that in the FTS treatment arm the arthritis was significantly attenuated (*P* < 0.001) as compared to CMC vehicle treated rats by both clinical scoring and ankle joint diameter measurements (Figures 1A,B). The clinical effects of FTS, with respect to arthritis severity and joint swelling, were of similar magnitude to the effects of MTX. Moreover, we also discovered that treatment with FTS as an add-on to MTX provided a very strong protective effect such as that the combined treatment almost completely inhibited the development of clinically evident arthritis (Figure 1A) and ankle joint swelling (Figure 1B). To further evaluate the effects of FTS on arthritis development, we examined ankle joint sections stained with hematoxylin and eosin from the various treatment arms (Figure 1C). Ankles of rats treated with adjuvant were given scores of 0–5 for bone resorption and inflammation, as previously described (44). The histological joint tissue sections from 0.5% CMC vehicle treated rats showed extensive infiltration with mononuclear cells (inflammation scores ranging from 4 to 5, *n* = 5), and significant bone destruction (bone resorption scores ranging from 4 to 5). In comparison, the sections from FTS treated rats showed less joint tissue infiltration by mononuclear cells (inflammation scores ranging from 2 to 3, *n* = 5), and less destruction of trabecular and cortical bone in the distal tibia (bone resorption scores ranging from 2 to 3). Moreover, the tissue sections from rats treated with FTS as an add-on to MTX showed only mild joint tissue infiltration with mononuclear cells (inflammation scores ranging from 2 to 3) and only rare areas of trabecular or cortical bone resorption not readily apparent on low magnification (average bone resorption scores of ≤2).

### FTS Inhibits the Upregulation of Pathogenic Serum Cytokines and Relevant Biomarkers

**Figure 2:**
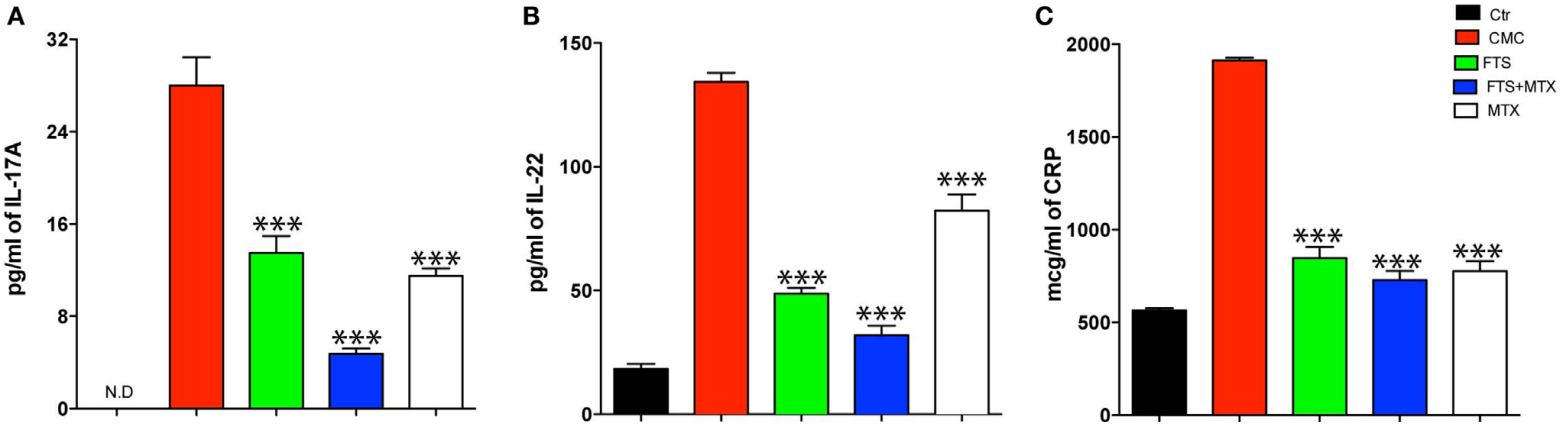
Farnesylthiosalicylic acid (FTS) treatment reduces serum Th17-cytokines and CRP levels during arthritis development. Sera from rats treated with carboxymethyl cellulose (CMC), FTS, FTS + MTX, or MTX was collected at Day 14 post adjuvant injection (day +3 from arthritis onset) and analyzed for **(A)** IL-17A, **(B)** IL-22, and **(C)** CRP levels by ELISA kits. Bars represent mean ± SD of triplicates from a representative experiment out of three performed (*n* = 4 rats analyzed per group). ND, not detectable. Statistical significance among groups was analyzed by the Student’s *t*-test (*P ≤ 0.05, **P ≤ 0.01, and ***P ≤ 0.001).

To investigate the immunomodulatory effect of FTS on the response to immunization with mTb containing CFA, we analyzed the levels of the key TH17 cell cytokines, IL-17A and IL-22, in serum samples collected at day 14 of the experiment. We found that treatment with FTS or MTX alone significantly reduced (*P* < 0.001) serum IL-17 levels compared to control CMC-treated arthritic rats (Figure 2A). Furthermore, combined treatment with FTS and MTX was extra potent in suppressing IL-17 upregulation, as compared to single agent therapy (*P* < 0.01). As predicted CFA immunization also induced a strong upregulation of serum IL-22 as compared to naïve healthy unimmunized littermate rats. The IL-22 response observed in CMC treated rats was significantly inhibited by either FTS or MTX therapy (*P* < 0.001) (Figure 2B). Interestingly, treatment with FTS alone or as an add-on to MTX had a more significant capacity to inhibit IL-22 production, as compared to MTX as a single agent (*P* < 0.01 for FTS and FTS + MTX vs. MTX).

In parallel, we analyzed the levels of serum CRP, a typical acute phase reactant protein in humans and in the rat (45). As expected, CFA injection induced a substantial increase in CRP levels in control CMC treated rats compared to naïve healthy littermate rats. Next, we found that FTS, MTX, and FTS as an add-on to MTX treatments equally and significantly reduced the arthritis-associated upregulation of serum CRP levels at day 14 of the experiments (Figure 2C).

### Characterizing the Immunomodulatory Effects of FTS on the *In Vivo* T Cell Response

**Figure 3:**
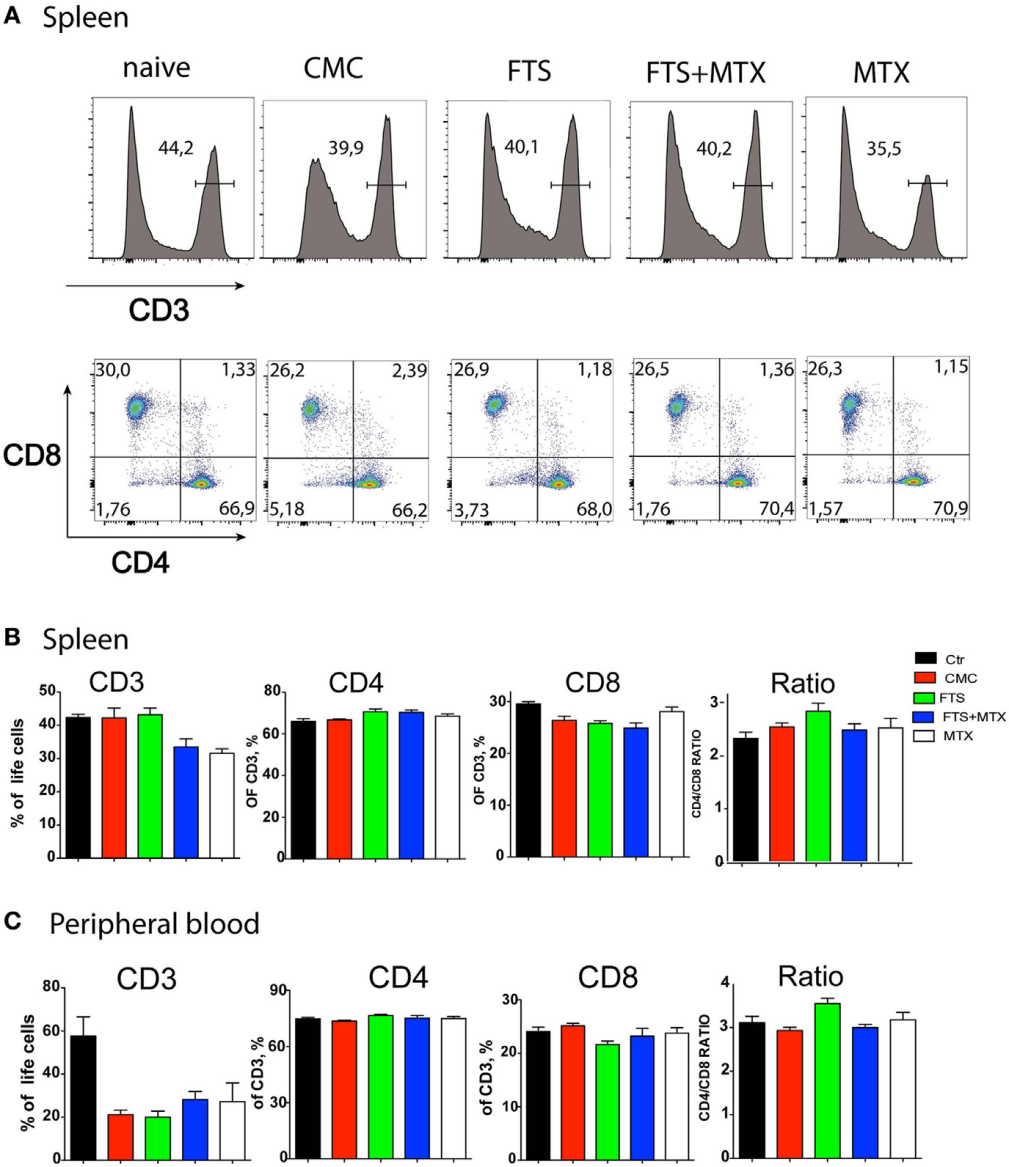
Effects of farnesylthiosalicylic acid (FTS) therapy on splenic and peripheral blood T cell populations. At study termination, single cell suspension of spleens **(A,B)** and peripheral blood **(C)** from the various groups of treated rats (*n* = 8 per group) were analyzed by flow cytometry for CD3^+^ CD4^+^, and CD8^+^ T cell frequencies. Samples were acquired on a FACSAria instrument (~50,000 single cell events per sample) and analyzed using the FlowJo software. The results shown represent a typical experiment out of >5 performed.

The use of this adjuvant model also offers an opportunity to study the pathological changes in a variety of tissues other than the joints. For example, the splenomegaly that develops as part of the systemic inflammation induced by CFA (40, 44). Thus, at study end, we could study the effects of the immunization and the various treatment protocols on the cellular immune response to CFA, principally analyzing by polychromatic flow cytometry the single cell suspension of spleens and peripheral blood samples for changes in CD4^+^ and CD8^+^ T cell percentages. Our results show that CFA immunization itself induced a significant decline (*P* < 0.05) in the percentage of circulating CD3^+^ T cells but not in splenic T cells. However, the various treatment regimens had only a marginal effect on the percentage of circulating and splenic CD3^+^ T cells. Moreover, we found that the various treatments had no significant effects on CD3^+^, CD4^+^, and CD8^+^ T cells percentage both in the spleen and the blood at study end. Nevertheless, we observed a trend (*P* = 0.08) for an increased CD4^+^ to CD8^+^ T cell ratio in the FTS therapy arm (Figures 3A–C).

**Figure 4:**
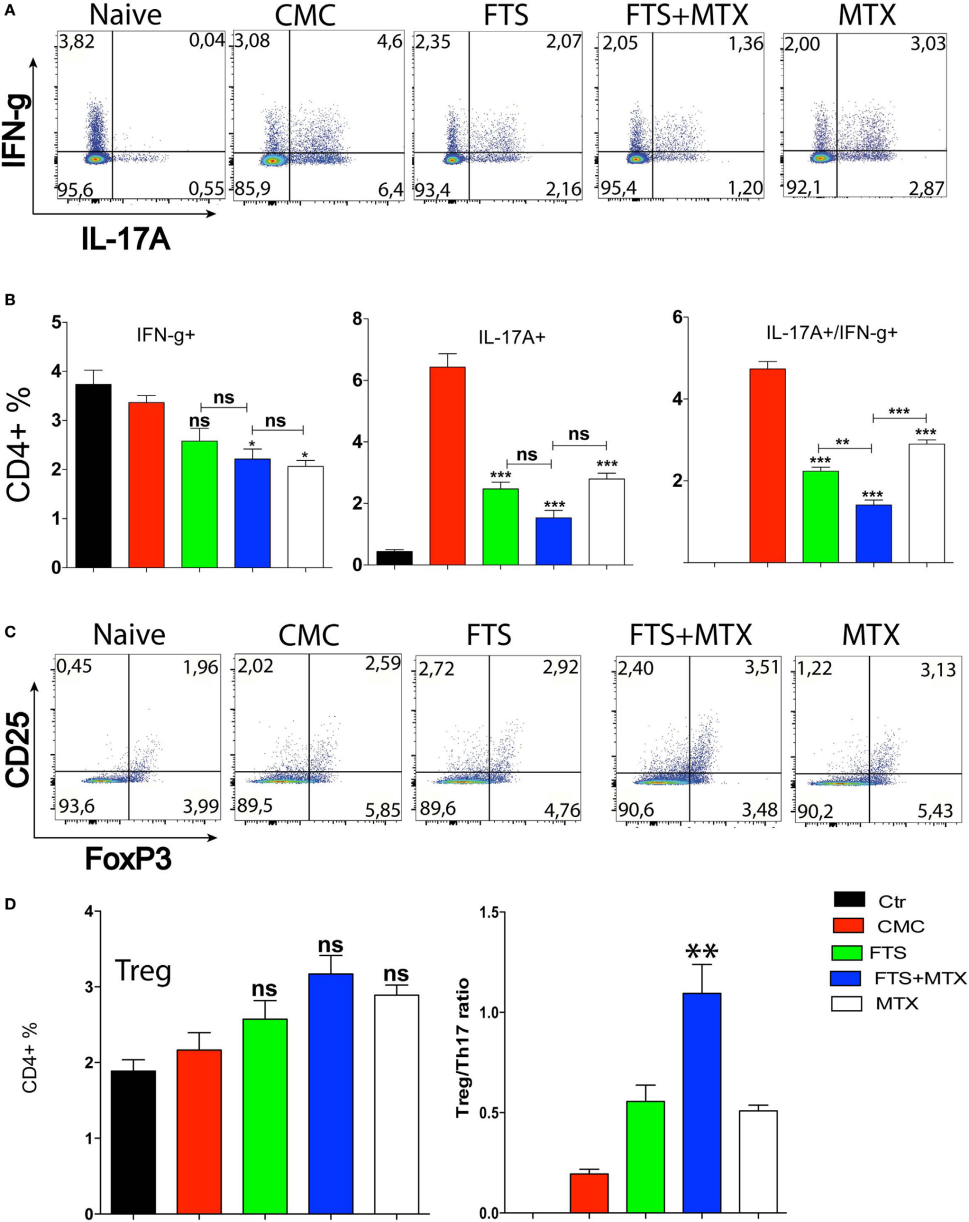
Immunomodulatory effect of farnesylthiosalicylic acid (FTS) treatment on the induction of Th1, Th17, and Treg subsets post CFA immunization. **(A)** Representative flow cytometry plots of intracellular staining for Th1 and Th17 cytokines producing CD4^+^ T cells. **(B)** Bars represent the percentage (mean ± SD) of the indicated Th-subsets in spleens of the various rat groups harvested at day 20 post CFA immunization (*n* = 5 per group). **(C)** Representative flow cytometry plots for Treg cells identification (CD3^+^CD4^+^FoxP3^+^ events). **(D)** Left hand Bars graph represent the percentage (mean ± SD) of Treg cells in the various rat groups (*n* = 5 per group). Right hand plot represents the Treg to Th17 ratio in these rats. Statistical significance was assessed by one way ANOVA with *post hoc* Bonferroni’s multiple comparison test (*P ≤ 0.05, **P ≤ 0.01, and ***P ≤ 0.001).

As both IL-17^+^ and IL-17^+^IFN-γ^+^ Th17-type cells have been shown to be instrumental for the pathogenesis of autoimmune responses (46), we analyzed the effects of FTS and other treatment protocols on the induction of IL-17 and IFN-γ expressing T cells in arthritic rats spleens by intracellular cytokine staining. Our data show that CFA immunization induced a significant induction of IL-17^+^ (~20-fold increase; *P* < 0.001) and of IL-17^+^IFN-γ^+^ Th17 cells (4.6 ± 0.34 vs. 0.04 ± 0.01, *P* < 0.01) compared to naive unimmunized rats. Moreover, FTS therapy alone as well as the other treatments all induced a significantly lower induction of pathogenic Th17 cells in the spleens of FTS treated rats compared to CMC vehicle treated arthritic rats *P* < 0.001. Interestingly, FTS as an add-on to MTX therapy induced a more significant reduction in the percentage of the highly pathogenic IL-17^+^IFN-γ^+^ T cells, as compared to either drug alone *P* < 0.01 (Figures 4A,B). Thus, the Th17 cell response data showed a positive and significant correlation with the clinical outcomes data of the various treatment protocols.

Next, we analyzed the effects of FTS and the different treatments on the induction of IFN-γ^+^ Th1 cells in the spleens. We found that, in contrast to the Th17 data, CFA immunization did not produce a significant increase in splenic Th1 cells percentage compared to naive unimmunized rats. Additionally, FTS therapy did not significantly reduce the induction of Th1 cells in the spleens of treated rats compared to CMC control arthritic rats. The combined FTS and MTX therapy only induced a marginally significant reduction in the percentage of Th1 cells as compared to FTS alone therapy (Figures 4A,B). Our results suggest that the improved clinical outcome produced by FTS therapy is less dependent on the inhibition of Th1 polarization.

As the balance between the inductions of antigen-specific pathogenic Th17 versus peripheral CD4^+^Foxp3^+^ regulatory T cells (Treg) can influence the outcome of the T cell response (47), we also determined the percentage of Treg cells in the spleens of the various rats. We found a statistically insignificant trend of increased Treg cells percentage in the spleens of FTS and FTS + MTX treated rats vs. CMC treated control arthritic rats (Figures 4C,D). Importantly, the ratio of Treg to Th17 cells was significantly increased (*P* < 0.01) when FTS and MTX therapy were combined, but not by the single drug therapy with either FTS or MTX (*P* = 0.07).

### FTS Therapy Modulates the Bhsp65-Induced Inflammatory Genes Expression Program of CD4^+^ T Cells from Treated Rats

The mycobacterial heat-shock protein 65 (Bhsp65), present in CFA, has been implicated in the immune-pathogenesis of AIA (41, 48). Hence, we used *in vitro* antigenic re-stimulation with Bhsp65 as an additional approach to study the effects of FTS therapy on the antigen-specific T cell response at the molecular level. Briefly, following immunization with CFA the animals were treated with FTS or CMC vehicle. At day +12 (onset of obvious clinical arthritis), the rats were euthanized, spleen, and draining superficial inguinal and para-aortic LN were harvested, and a single-cell suspension of a mixture of spleen and LN cells was prepared. Subsequently, the cells were cultured for four days with or without recombinant Bhsp65 protein (5 µg/ml), as previously described (49) and total RNA was purified at termination of the experiment.

To gain a more comprehensive insight into the gene networks that are associated with the therapeutic action of Ras-inhibitor in AIA, we analyzed by GeneChip^®^ microarrays the changes in global gene expression (primarily mRNAs) associated with FTS treatment. Thus, at day +12, we purified CD4^+^ T cells from the spleen and relevant draining LNs of FTS treated and control rats, re-stimulated them with Bhsp65 *in vitro* and then cultured them for additional 4 days. At the end of experiment, total RNA was purified and used for the downstream transcriptome analysis.

**Figure 5:**
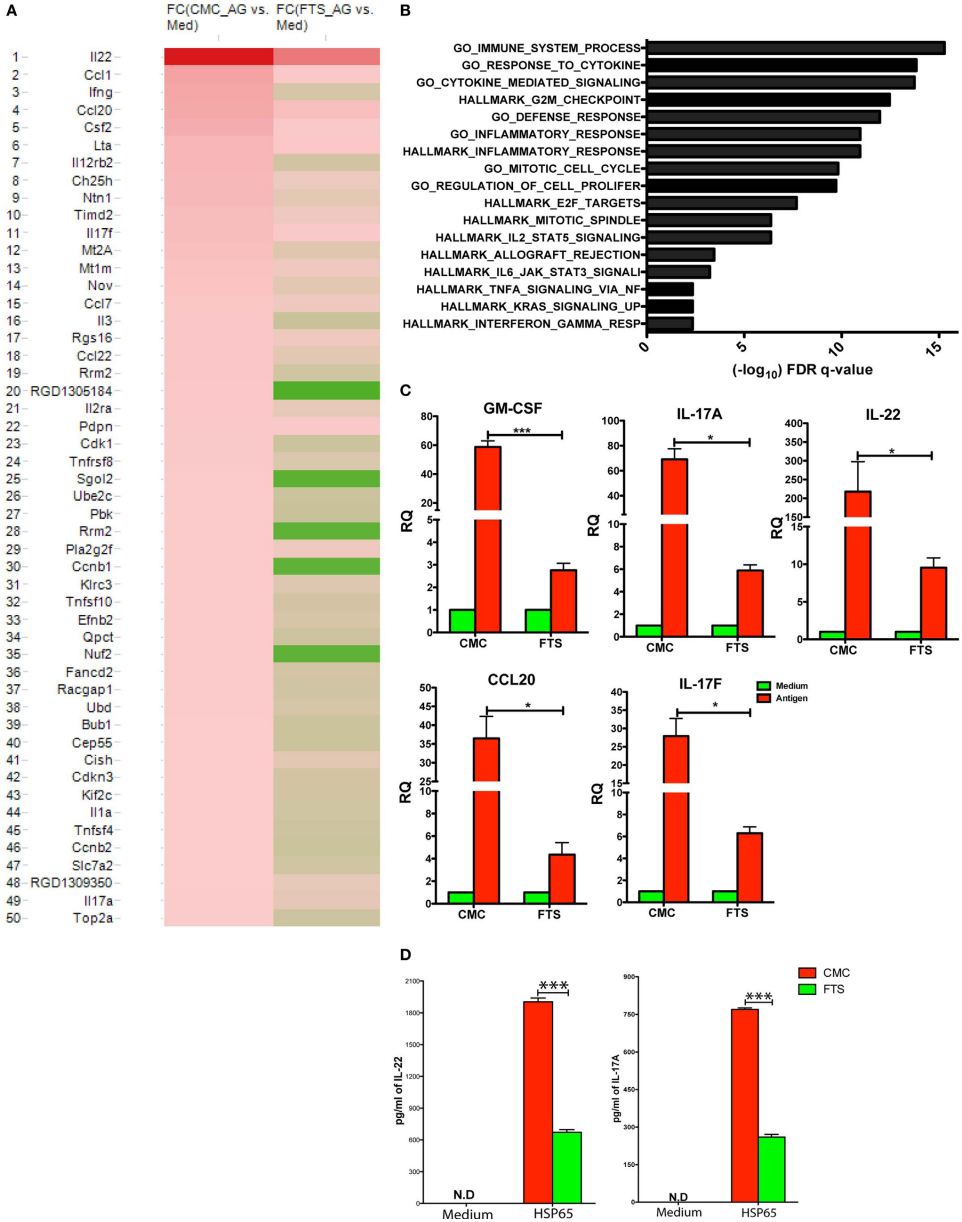
Farnesylthiosalicylic acid (FTS) therapy down modulates the Bhsp65-induced transcription of multiple genes tightly linked to inflammatory and immune processes. Following CFA-immunization, rats were treated with carboxymethyl cellulose (CMC) or FTS starting from day +1 post AIA induction. Single cell suspensions from draining LNs and spleen of treated rats (harvested on day +14 post disease induction) were stimulated with Bhsp65 (5 µg/ml) or control PBS and cultured for additional 72 h. At the end of cultures, CD4^+^ T cells were isolated and high quality total RNA was extracted from the purified cells. **(A)** Heat map analysis depicting the expression matrix of the 50 genes significantly induced by Bhsp65 stimulation (>2FC, FDR *q* < 0.05) in the CMC control group as compared to their expression in FTS treated group (*n* = 2 per group). In this two-color heat map, the brightest red and green colors represent the top up- or down-regulated mRNAs, respectively. **(B)** Next, we computed using the GSEA software web site v6.1 software, the overlaps between our gene list and relevant validated gene sets. Bars represent the FDR *q*-value (cutoff of <0.05) for the statistical significance of the overlap between the annotated Gene set and our list. **(C)** Purified total RNA was analyzed by quantitative PCR for the relative mRNA expression (normalized to Actb) of five inflammatory response genes (Ccl20, Il22, Il17A, Il17F, and Csf2) induced by Bhsp65 re-stimulation of CD4^+^ T cells treated *in vivo* with CMC vs. FTS. **(D)** Supernatants from the same cultures were tested by ELISA kits for levels of secreted 17A and IL-22 by readymade ELISA kits. Bar graphs depict mean ± SD, and statistical significance was analyzed by Student’s *t*-test (*P ≤ 0.05, **P ≤ 0.01, and ***P ≤ 0.001). Data shown are representative of a typical experiment out of *n* = 3.

The bioinformatics analysis of the various CD4^+^ T cell samples showed that the antigenic re-stimulation with Bhsp65, as expected, induced a robust upregulation of multiple (*n* = 50) genes in CD4^+^ T cells (Figure 5A). As seen in the plotted heat map, depicting the signal intensity of the list of Bhsp65-induced and differentially expressed genes (transcripts with ≥twofold change; 2FC) in CD4^+^ T cells of CMC as well as FTS treated rats. The plot shows a widespread FTS-dependent reduction in the intensity of the Bhsp65-induced transcription of a large percentage of these 50 genes (*P* < 0.001, by chi-square test). Notably, this list includes a large number of recognized immune response genes, such as genes encoding pro-inflammatory cytokines (e.g., Il22, Il17a/f, Ifng, Csf2/GM-CSF, Lta, and Il1a).

Next, to determine, in unbiased manner, the biological processes and molecular functions that mediate the therapeutic effect of FTS, we performed additional in-depth bioinformatics analysis. Thus, we computed the overlaps between our gene list and relevant annotated gene sets within the HALLMARK and gene ontology (GO) collections of the Molecular Signatures Database of the Broad Institute (Massachusetts Institute of Technology) using the GSEA software web site v6.1 (50).

By this analysis, we determined that the list of genes upregulated significantly (>twofold change, FDR *q* < 0.05) following Bhsp65 re-stimulation of CD4^+^ T cells isolated from CMC treated control rats exhibited significant overlap with a large number of curated relevant immune response and cell proliferation gene sets. The top overlapping annotated gene sets included: (i) cytokine activity (GO); immune system processes (GO); G2M_checkpoint (HALLMARK); inflammatory response (HALLMARK); response to tumor necrosis factor (GO); response to IFN-gamma (GO); positive regulation of cell proliferation (GO); IL6_JAK_STAT3_signaling (HALLMARK); K-RAS signaling up (HALLMARK); and others (see also Figure 5B).

To validate our GeneChip^®^ data, we analyzed by qPCR the relative quantity of mRNA transcripts of five highly relevant inflammatory response genes differentially induced by Bhsp65 re-stimulation in CMC vs. FTS treated CD4^+^ T cells (ccl20, il22, il17A, il17F, and Csf2). As shown in Figure 5C, the qPCR data confirmed that the induced transcription of all these genes, during the recall response of TH cells to Bhsp65, was significantly inhibited by FTS therapy (*P* < 0.05, by *t*-test). In agreement with our gene transcription data, we also detected by ELISA a strong induction of IL-17A and IL-22 protein expression following *in vitro* Bhsp65 re-stimulation of control CD4^+^ T cells (Figure 5D), whereas this antigen-dependent induction of IL-17A and IL-22 secretion was strongly inhibited in equivalent cultures of FTS treated rats (*P* < 0.001, by *t*-test).

### The FTS Derivative 5-Fluoro-FTS (F-FTS) Shows a Higher Therapeutic Effect than FTS

**Figure 6:**
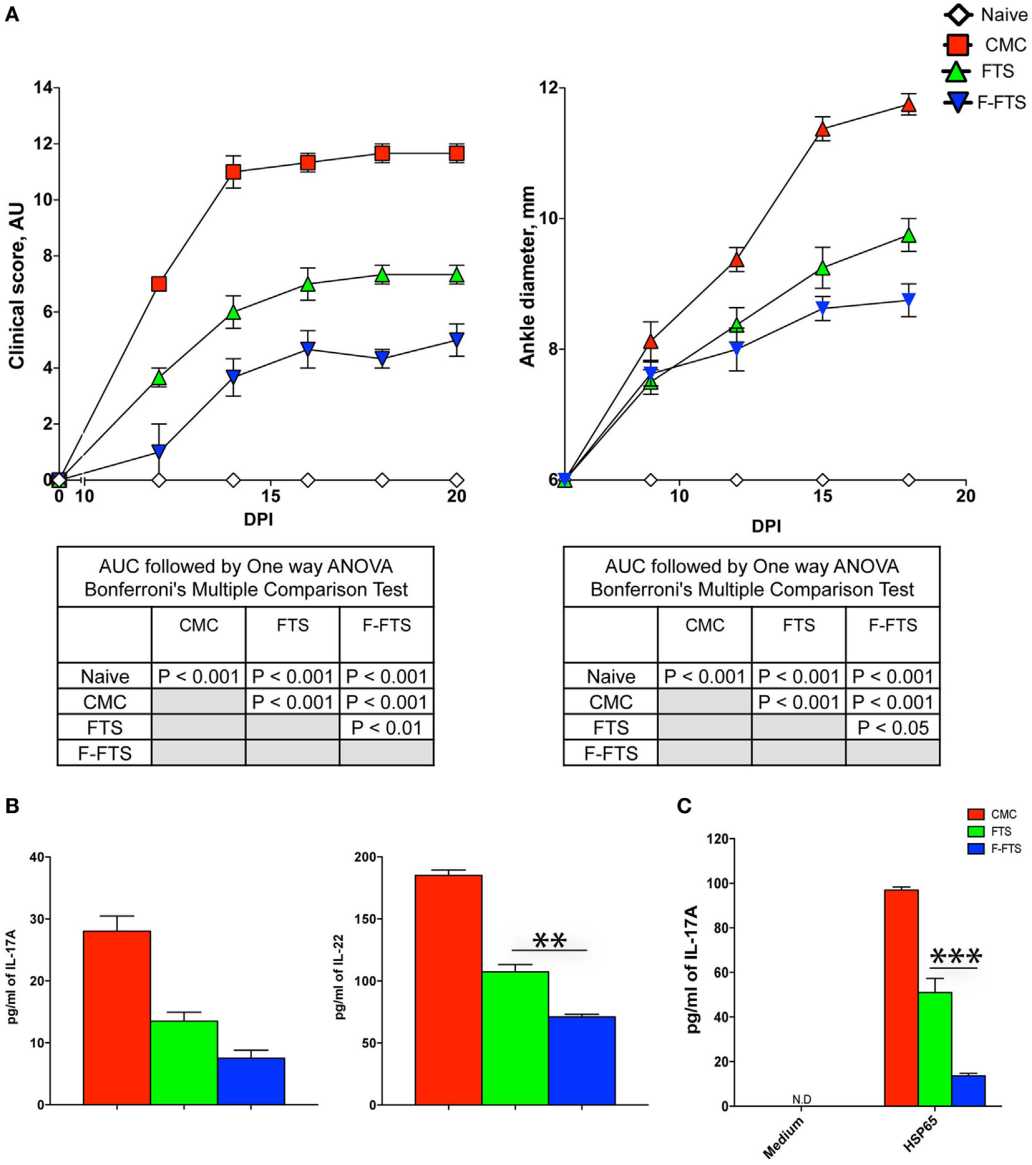
Fluoro-FTS (F-FTS) is significantly more effective than farnesylthiosalicylic acid (FTS) in attenuating multiple outcome measures of AIA. **(A)** Rats immunized with CFA were treated starting day +1 with either oral FTS (100 mg/kg), F-FTS (60 mg/kg), or 0.5% carboxymethyl cellulose (CMC) vehicle solution. Arthritis severity was graded by clinical scores (left panel) and ankle diameter (right panel). Statistical analysis of the AUC of the effect of the treatments on the clinical outcomes was done by one way ANOVA with *post hoc* Bonferroni’s multiple comparison test, and the results are summarized in the table insets. **(B)** Sera from rats treated with either CMC, FTS, or F-FTS (*n* = 5 per group) were collected at day +14 and analyzed for IL-17A and IL-22 levels by readymade ELISA kits. **(C)** In paralleled, single cell suspensions from draining LNs and spleen of the various rats were stimulated with Bhsp65 (5 µg/ml) of or vehicle and cultured for additional 72 h. At culture termination, we analyzed the supernatants for the levels of Bhsp65-induced 17A secretion by ELISA. One representative experiments out of two performed. Bar graphs depict mean ± SD. Statistical significance was assessed by the Student’s *t*-test (*P ≤ 0.05, **P ≤ 0.01, and ***P ≤ 0.001).

Fluoro-FTS, although presumed to have a rather similar mechanism of action as the parent compound, has been previously shown to be a more potent inhibitor of oncogenic Ras signaling as compared to FTS (51). Moreover, F-FTS treatment was previously shown to strongly attenuate the development of type-1 diabetes in NOD mice (36). However, the *in vivo* therapeutic efficacy of F-FTS and FTS has not been previously compared. Thus, we designed a set of experiments to investigate the relative therapeutic efficacy of F-FTS compared to FTS. The data from these studies demonstrate that both F-FTS and FTS therapy following CFA immunization produced a significant attenuation of disease development and severity, as assessed by the clinical score and ankle joint diameter measurements, as compared to CMC treated control rats. Importantly, the therapeutic efficacy of F-FTS was significantly superior compared to FTS (Figure 6A). Next, we show that F-FTS therapy produced a robust reduction of serum IL-17 and IL-22 levels post CFA immunization, as compared to control immunized rats (Figure 6B). Moreover, the inhibitory effect on IL-22 induction was more pronounced with F-FTS vs. FTS therapy (*P* < 0.001, by *t*-test).

Next, we analyzed the effect of F-FTS therapy on the induction of antigen-specific Th17 cells. Thus, as already detailed above we employed *in vitro* antigenic re-stimulation with Bhsp65 to test this question. Our results demonstrated that *in vivo* therapy with either F-FTS or FTS was coupled with significant inhibition of IL-17 production during the recall response of Bhsp65-specific T cells compared to the robust IL-17 recall response in control CMC treated rats cultures (*P* < 0.001 for both drugs vs. CMC, by one-way ANOVA). Moreover, as shown in Figure 6C, F-FTS was a more potent inhibitor compared to FTS (*P* < 0.05), which positively correlated with its superior therapeutic efficacy.

## Discussion

In this study, we further demonstrate the therapeutic efficacy of the orally active small-molecule inhibitors of PM-localization of Ras GTPases, FTS, and its more potent derivative F-FTS, in the rat AIA model of RA. Therapy with either FTS or F-FTS significantly reduced ankle swelling and clinical arthritis scores. Moreover, the therapeutic efficacy of Ras inhibitors was comparable to that of the mainstay disease modifying anti-rheumatic drug (DMARD), MTX. Importantly, FTS treatment as an add-on to MTX therapy resulted in a robust attenuation of arthritis development. The histopathological assessment of the ankle joints confirmed that FTS treatment reduced ankle joint inflammation and bone resorption. The clinical efficacy of the Ras inhibitors strongly correlated with reduced serum levels of IL-17 and IL-22 together with a reduced recall Th17-response to Bhsp65-antigen stimulation in splenocytes from FTS or F-FTS treated rats. Moreover, the bioinformatics of the gene microarrays further demonstrated the wide-ranging efficacy of FTS to down-modulate the TCR activation induced transcription of a large set of canonical pro-inflammatory genes in effector CD4^+^ T cells following *in vitro* Bhsp65 re-challenging.

Initial findings in a previous study showed that FTS treatment reduced the clinical arthritis score in the AIA model, and that this treatment was associated with reduced levels of various pro-inflammatory cytokines (IFN-γ, TNF-α, IL-6, and IL-17) in the serum at study end. Our current work was designed to obtain new and pertinent data on the effects of oral Ras inhibitors on the pathogenic T cell response to CFA, and more specifically to the arthritogenic mycobacterial antigen, Bhsp65. We also provide new insight on the molecular effects of *in vivo* Ras-signaling blockade on the transcriptomes of Bhsp65-specific CD4^+^ T cells. In the present study, we also tested in parallel the therapeutic effects of F-FTS (FTS derivative) and of FTS as an add-on to the drug MTX.

The Ras family of GTPases play an important role in signaling nodes downstream to TCR and CD28 activation (52). Perturbation of K-Ras-signaling lowers the threshold for TCR activation and thus may support abnormal responses to autoantigens (10). For example, somatic mutation in NRAS or KRAS genes in hematopoietic cells can cause a rare autoimmune disorder, characterized by lymphadenopathy and splenomegaly due to abnormal expansion of lymphocytes (53, 54). Therefore, our working hypothesis was that our Ras inhibitors might chiefly target the T cell response to CFA injection. As extensive research in murine arthritis models strongly indicates a central role for the Th17-type cells (55, 56), we focused on the effects of FTS on the induction of the Th17 response.

Our data indeed show that CFA injection was linked to a robust induction of Th17 cells, and that importantly FTS therapy reduced the frequency of Th17 cells in relevant lymphoid tissues (draining LNs and spleen). The two chief CD4^+^ T cells subsets suppressed by FTS were IL17^+^ (classical Th17 cells) and IL-17^+^IFN-γ^+^ “double positive” cells. The inhibitory effect on the induction of these IL-17 producing T cell subsets was comparable to the effect of MTX therapy, while the combined FTS and MTX was significantly more potent in this regard. In contrast, FTS therapy had no significant effect on the induction IFN-γ^+^CD4^+^ T cells (classical Th1 cells). Accordingly, we also detected reduced serum IL-17 levels at arthritis onset in FTS treated rats. Moreover, FTS and F-FTS therapy strongly attenuated Bhsp65-induced IL-17 production by CD4^+^ T cells (both per ELISA and per GeneChip^®^ array data).

Although the prototype Th17 cell cytokines, IL-17A/F, have been strongly linked to autoimmunity in multiple animal models, there has been recent evidence that a fraction of Th17 cells also co-express IFN-γ at the site of inflammation with reported functional consequences (57). Our data are the first work that shows that CFA immunization induces significant expansion of both classical Th17 cells and Th cells with a Th17/Th1 “double phenotype.” Moreover, we clearly determine by intracellular cytokine staining that FTS treatment strongly targets the expansion of this unique and likely pathogenic T cell subset in relevant lymphoid tissues.

By functional enrichment analysis of the GeneChip^®^ array data, utilizing the GSEA platform (50), we identified a list of validated immune/inflammatory response genes that are “silenced” by FTS therapy specifically in pathogenic CD4^+^ Th cells responding to Bhsp65. For example, besides the classical Th17 cytokine genes (*Il7* and *Il22*), we also identified that FTS targeted other pro-inflammatory cytokine genes (e.g., *Ifng*, Cfs2, Lta, Il1a) as well as chemokines genes that are chemotactic for effector T cells, monocytes, and dendritic cells (Ccl1, Ccl20, Ccl7, and Ccl22). Thus, these data allowed us to discover additional interesting genes, molecular processes, and biological pathways that expand our insight into the mechanism of action of FTS.

The Th17-allied cytokine IL-22 has been shown to play a pathogenic role in murine models of arthritis, particularly in promoting the early inflammatory responses to CFA and enhancing cartilage and bone damage (58–60). Moreover, levels of IL-22 and Th22 cells are increased in the synovial tissue and in the in the blood of RA patients, and correlate with disease activity (61, 62). Importantly, IL-22 mainly secreted locally by T cells, can induce synovial fibroblasts’ proliferation and RANKL (Receptor Activator of NF-κB Ligand) expression, and consequently promote their differentiation into osteoclasts (63). Our data clearly show that *in vivo* treatment with Ras inhibitors strongly attenuated the CFA-induced upregulation of serum IL-22 as well as the transcription and secretion of IL-22 by purified effector CD4^+^ T cells re-challenged *in vitro* with the mycobacterial antigen, Bhsp65.

Our novel finding that the combined MTX and FTS treatment was more potent, both by clinical and laboratory outcome measures, as compared to monotherapy with either drug alone in AIA, is highly relevant to RA treatment. MTX is commonly referred to as the cornerstone of modern RA therapy as a large percentage of patients are effectively treated with MTX alone or in combination with other drugs. Moreover, the majority of anti-RA biologics are more effective as a combination therapy with a conventional DMARD, predominantly MTX, as compared to biologics monotherapy (64). Thus, our findings strongly fit into this clinical concept: i.e., combination therapy with MTX is superior to monotherapy with the targeted-synthetic immunomodulatory compound, FTS.

Additionally, we performed for the first time a “head-to-head” comparison of the therapeutic efficacy of F-FTS vs. FTS. Our findings demonstrate that F-FTS has a superior therapeutic efficacy compared to its parent compound, FTS, at a lower dose (60 mg/kg compared to 100 mg/kg, respectively). Moreover, our ELISA data show, in agreement with the clinical outcome data, that F-FTS was a more potent inhibitor of the *in vivo* Th17 response to CFA injection. Thus, the results from the latter set of studies add to the translational impact of the paper.

In conclusion, FTS and F-FTS, two targeted synthetic Ras-GTPases inhibitors, exhibited a potent immunomodulatory effect in the classical animal model of arthritis, AIA, which was further enhanced by combination therapy with MTX. The therapeutic effect was coupled with *in vivo* inhibition of CFA-induced generation of Th17-polarized, IL-17 and IL-22 secreting, lymphocytes. Thus, Ras-signaling-blockade is a promising novel therapeutic approach for RA, justifying further preclinical evaluation of these compounds in RA patients.

## Ethics Statement

All animal experiments were conducted in accordance with relevant laws of the state of Israel and guidelines of the Tel-Aviv University and approved by the Institutional Animal Care and Use Committee (Approval # L-14-018), and by the USAMRMC Animal Care and Use Review Office (protocol PR130028.03).

## Author Contributions

MZ, VM-M, YK, and IG designed the research and analyzed data; MZ, VM-M, EV, GE-S, and JJ-H performed the research; YK and IB contributed new reagents/analytic tools; and MZ and IG wrote the paper. IG approved the final version of the manuscript.

## Conflict of Interest Statement

The authors declare that the research was conducted in the absence of any commercial or financial relationships that could be construed as a potential conflict of interest.

## References

[1] Klarenbeek NB Kerstens PJ Huizinga TW Dijkmans BA Allaart CF Recent advances in the management of rheumatoid arthritis. BMJ (2010) 341:c6942 10.1136/bmj.c6942 21177351

[2] Yang J Sundrud MS Skepner J Yamagata T. Targeting Th17 cells in autoimmune diseases. Trends Pharmacol Sci (2014) 35:493–500. 10.1016/j.tips.2014.07.006 25131183

[3] Elson CO Cong Y Weaver CT Schoeb TR McClanahan TK Fick RB Monoclonal anti-interleukin 23 reverses active colitis in a T cell-mediated model in mice. Gastroenterology (2007) 132:2359–70. 10.1053/j.gastro.2007.03.104 17570211

[4] Langrish CL Chen Y Blumenschein WM Mattson J Basham B Sedgwick JD IL-23 drives a pathogenic T cell population that induces autoimmune inflammation. J Exp Med (2005) 201:233–40. 10.1084/jem.20041257 15657292 PMC2212798

[5] Nakae S Nambu A Sudo K Iwakura Y. Suppression of immune induction of collagen-induced arthritis in IL-17-deficient mice. J Immunol (2003) 171:6173–7. 10.4049/jimmunol.171.11.6173 14634133

[6] Bush KA Farmer KM Walker JS Kirkham BW. Reduction of joint inflammation and bone erosion in rat adjuvant arthritis by treatment with interleukin-17 receptor IgG1 Fc fusion protein. Arthritis Rheum (2002) 46:802–5. 10.1002/art.10173 11920418

[7] Fields PE Gajewski TF Fitch FW. Blocked Ras activation in anergic CD4+ T cells. Science (1996) 271:1276–8. 10.1126/science.271.5253.1276 8638108

[8] Schwartz RH T cell anergy. Annu Rev Immunol (2003) 21:305–34. 10.1146/annurev.immunol.21.120601.141110 12471050

[9] Zha Y Marks R Ho AW Peterson AC Janardhan S Brown I T cell anergy is reversed by active Ras and is regulated by diacylglycerol kinase-alpha. Nat Immunol (2006) 7:1166–73. 10.1038/ni1206-1343a 17028589

[10] Singh K Deshpande P Li G Yu M Pryshchep S Cavanagh M K-RAS GTPase- and B-RAF kinase-mediated T-cell tolerance defects in rheumatoid arthritis. Proc Natl Acad Sci U S A (2012) 109:E1629–37. 10.1073/pnas.1117640109 22615393 PMC3382540

[11] Singh K Deshpande P Pryshchep S Colmegna I Liarski V Weyand CM ERK-dependent T cell receptor threshold calibration in rheumatoid arthritis. J Immunol (2009) 183:8258–67. 10.4049/jimmunol.0901784 20007589 PMC2828269

[12] Rainy N Chetrit D Rouger V Vernitsky H Rechavi O Marguet D H-Ras transfers from B to T cells via tunneling nanotubes. Cell Death Dis (2013) 4:e726. 10.1038/cddis.2013.245 23868059 PMC3730418

[13] Rechavi O Goldstein I Vernitsky H Rotblat B Kloog Y. Intercellular transfer of oncogenic H-Ras at the immunological synapse. PLoS One (2007) 2:e1204. 10.1371/journal.pone.0001204 18030338 PMC2065899

[14] Vernitsky H Rechavi O Rainy N Besser MJ Nagar M Schachter J Ras oncoproteins transfer from melanoma cells to T cells and modulate their effector functions. J Immunol (2012) 189:4361–70. 10.4049/jimmunol.1200019 23028055

[15] Bos JL Ras oncogenes in human cancer: a review. Cancer Res (1989) 49:4682–9. 2547513

[16] Cox AD Der CJ. Ras history: the saga continues. Small GTPases (2010) 1:2–27. 10.4161/sgtp.1.1.12178 21686117 PMC3109476

[17] Blum R Cox AD Kloog Y. Inhibitors of chronically active ras: potential for treatment of human malignancies. Recent Pat Anticancer Drug Discov (2008) 3:31–47. 10.2174/157489208783478702 18289122

[18] Kloog Y Cox AD. Ras inhibitors: potential for cancer therapeutics. Mol Med Today (2000) 6:398–402. 10.1016/S1357-4310(00)01789-5 11006529

[19] Kloog Y Cox AD. Prenyl-binding domains: potential targets for Ras inhibitors and anti-cancer drugs. Semin Cancer Biol (2004) 14:253–61. 10.1016/j.semcancer.2004.04.004 15219618

[20] Kloog Y Cox AD Sinensky M. Concepts in Ras-directed therapy. Expert Opin Investig Drugs (1999) 8:2121–40. 10.1517/13543784.8.12.2121 11139843

[21] Arozarena I Calvo F Crespo P. Ras, an actor on many stages: posttranslational modifications, localization, and site-specified events. Genes Cancer (2011) 2:182–94. 10.1177/1947601911409213 21779492 PMC3128639

[22] Choy E Chiu VK Silletti J Feoktistov M Morimoto T Michaelson D Endomembrane trafficking of ras: the CAAX motif targets proteins to the ER and Golgi. Cell (1999) 98:69–80. 10.1016/S0092-8674(00)80607-8 10412982

[23] Hancock JF Magee AI Childs JE Marshall CJ. All ras proteins are polyisoprenylated but only some are palmitoylated. Cell (1989) 57:1167–77. 10.1016/0092-8674(89)90054-8 2661017

[24] Gana-Weisz M Haklai R Marciano D Egozi Y Ben-Baruch G Kloog Y. The Ras antagonist S-farnesylthiosalicylic acid induces inhibition of MAPK activation. Biochem Biophys Res Commun (1997) 239:900–4. 10.1006/bbrc.1997.7582 9367867

[25] Marom M Haklai R Ben-Baruch G Marciano D Egozi Y Kloog Y. Selective inhibition of Ras-dependent cell growth by farnesylthiosalisylic acid. J Biol Chem (1995) 270:22263–70. 10.1074/jbc.270.38.22263 7673206

[26] Elad-Sfadia G Haklai R Ballan E Gabius HJ Kloog Y. Galectin-1 augments Ras activation and diverts Ras signals to Raf-1 at the expense of phosphoinositide 3-kinase. J Biol Chem (2002) 277:37169–75. 10.1074/jbc.M205698200 12149263

[27] Farin K Schokoroy S Haklai R Cohen-Or I Elad-Sfadia G Reyes-Reyes ME Oncogenic synergism between ErbB1, nucleolin, and mutant Ras. Cancer Res (2011) 71:2140–51. 10.1158/0008-5472.CAN-10-2887 21257709

[28] Paz A Haklai R Elad-Sfadia G Ballan E Kloog Y. Galectin-1 binds oncogenic H-Ras to mediate Ras membrane anchorage and cell transformation. Oncogene (2001) 20:7486–93. 10.1038/sj.onc.1204950 11709720

[29] Rotblat B Niv H Andre S Kaltner H Gabius HJ Kloog Y. Galectin-1(L11A) predicted from a computed galectin-1 farnesyl-binding pocket selectively inhibits Ras-GTP. Cancer Res (2004) 64:3112–8. 10.1158/0008-5472.CAN-04-0026 15126348

[30] Ashery U Yizhar O Rotblat B Elad-Sfadia G Barkan B Haklai R Spatiotemporal organization of Ras signaling: rasosomes and the galectin switch. Cell Mol Neurobiol (2006) 26:471–95. 10.1007/s10571-006-9059-3 16691442 PMC11520644

[31] Charette N De Saeger C Horsmans Y Leclercq I Starkel P. Salirasib sensitizes hepatocarcinoma cells to TRAIL-induced apoptosis through DR5 and survivin-dependent mechanisms. Cell Death Dis (2013) 4:e471. 10.1038/cddis.2012.200 23348585 PMC3563988

[32] Charette N De Saeger C Lannoy V Horsmans Y Leclercq I Starkel P. Salirasib inhibits the growth of hepatocarcinoma cell lines in vitro and tumor growth in vivo through ras and mTOR inhibition. Mol Cancer (2010) 9:256. 10.1186/1476-4598-9-256 20860815 PMC2955616

[33] Schneider-Merck T Borbath I Charette N De Saeger C Abarca J Leclercq I The Ras inhibitor farnesylthiosalicyclic acid (FTS) prevents nodule formation and development of preneoplastic foci of altered hepatocytes in rats. Eur J Cancer (2009) 45:2050–60. 10.1016/j.ejca.2009.04.014 19427195

[34] Aizman E Mor A Chapman J Assaf Y Kloog Y. The combined treatment of Copaxone and Salirasib attenuates experimental autoimmune encephalomyelitis (EAE) in mice. J Neuroimmunol (2010) 229:192–203. 10.1016/j.jneuroim.2010.08.022 20869125

[35] Karussis D Abramsky O Grigoriadis N Chapman J Mizrachi-Koll R Niv H The Ras-pathway inhibitor, S-trans-trans-farnesylthiosalicylic acid, suppresses experimental allergic encephalomyelitis. J Neuroimmunol (2001) 120:1–9. 10.1016/S0165-5728(01)00385-X 11694313

[36] Aizman E Mor A George J Kloog Y. Ras inhibition attenuates pancreatic cell death and experimental type 1 diabetes: possible role of regulatory T cells. Eur J Pharmacol (2010) 643:139–44. 10.1016/j.ejphar.2010.06.029 20599916

[37] Oron T Elad-Sfadia G Haklai R Aizman E Brazowski E Kloog Y Prevention of induced colitis in mice by the ras antagonist farnesylthiosalicylic acid. Dig Dis Sci (2012) 57:320–6. 10.1007/s10620-011-1880-y 21901261

[38] Katzav A Kloog Y Korczyn AD Niv H Karussis DM Wang N Treatment of MRL/lpr mice, a genetic autoimmune model, with the Ras inhibitor, farnesylthiosalicylate (FTS). Clin Exp Immunol (2001) 126:570–7. 10.1046/j.1365-2249.2001.01674.x 11737078 PMC1906212

[39] Aizman E Blacher E Ben-Moshe O Kogan T Kloog Y Mor A. Therapeutic effect of farnesylthiosalicylic acid on adjuvant-induced arthritis through suppressed release of inflammatory cytokines. Clin Exp Immunol (2014) 175:458–67. 10.1111/cei.12235 24215151 PMC3927906

[40] Bendele A Animal models of rheumatoid arthritis. J Musculoskelet Neuronal Interact (2001) 1:377–85. 15758488

[41] Mia MY Kim EY Satpute SR Moudgil KD. The dynamics of articular leukocyte trafficking and the immune response to self heat-shock protein 65 influence arthritis susceptibility. J Clin Immunol (2008) 28:420–31. 10.1007/s10875-008-9205-4 18481159

[42] van Eden W Thole JE van der Zee R Noordzij A van Embden JD Hensen EJ Cloning of the mycobacterial epitope recognized by T lymphocytes in adjuvant arthritis. Nature (1988) 331:171–3. 10.1038/331171a0 2448638

[43] Cobelens PM Heijnen CJ Nieuwenhuis EE Kramer PP van der Zee R van Eden W Treatment of adjuvant-induced arthritis by oral administration of mycobacterial Hsp65 during disease. Arthritis Rheum (2000) 43:2694–702. 10.1002/1529-0131(200012)43:12<2694::AID-ANR9>3.0.CO;2-E 11145027

[44] Bendele A McComb J Gould T McAbee T Sennello G Chlipala E Animal models of arthritis: relevance to human disease. Toxicol Pathol (1999) 27:134–42. 10.1177/019262339902700125 10367688

[45] Giffen PS Turton J Andrews CM Barrett P Clarke CJ Fung KW Markers of experimental acute inflammation in the Wistar Han rat with particular reference to haptoglobin and C-reactive protein. Arch Toxicol (2003) 77:392–402. 10.1007/s00204-003-0458-7 12669191

[46] Tabarkiewicz J Pogoda K Karczmarczyk A Pozarowski P Giannopoulos K. The role of IL-17 and Th17 lymphocytes in autoimmune diseases. Arch Immunol Ther Exp (2015) 63:435–49. 10.1007/s00005-015-0344-z 26062902 PMC4633446

[47] Noack M Miossec P Th17 and regulatory T cell balance in autoimmune and inflammatory diseases. Autoimmun Rev (2014) 13:668–77. 10.1016/j.autrev.2013.12.004 24418308

[48] Huang MN Yu H Moudgil KD. The involvement of heat-shock proteins in the pathogenesis of autoimmune arthritis: a critical appraisal. Semin Arthritis Rheum (2010) 40:164–75. 10.1016/j.semarthrit.2009.10.002 19969325 PMC3390779

[49] Yu H Lu C Tan MT Moudgil KD. Comparative antigen-induced gene expression profiles unveil novel aspects of susceptibility/resistance to adjuvant arthritis in rats. Mol Immunol (2013) 56:531–9. 10.1016/j.molimm.2013.05.230 23911410 PMC3783567

[50] Subramanian A Tamayo P Mootha VK Mukherjee S Ebert BL Gillette MA Gene set enrichment analysis: a knowledge-based approach for interpreting genome-wide expression profiles. Proc Natl Acad Sci U S A (2005) 102:15545–50. 10.1073/pnas.0506580102 16199517 PMC1239896

[51] Aharonson Z Gana-Weisz M Varsano T Haklai R Marciano D Kloog Y. Stringent structural requirements for anti-Ras activity of S-prenyl analogues. Biochim Biophys Acta (1998) 1406:40–50. 10.1016/S0925-4439(97)00077-X 9545527

[52] Chen L Flies DB. Molecular mechanisms of T cell co-stimulation and co-inhibition. Nat Rev Immunol (2013) 13:227–42. 10.1038/nri3405 23470321 PMC3786574

[53] Niemela JE Lu L Fleisher TA Davis J Caminha I Natter M Somatic KRAS mutations associated with a human nonmalignant syndrome of autoimmunity and abnormal leukocyte homeostasis. Blood (2011) 117:2883–6. 10.1182/blood-2010-07-295501 21079152 PMC3062298

[54] Oliveira JB The expanding spectrum of the autoimmune lymphoproliferative syndromes. Curr Opin Pediatr (2013) 25:722–9. 10.1097/MOP.0000000000000032 24240292 PMC4435794

[55] Miossec P Kolls JK. Targeting IL-17 and TH17 cells in chronic inflammation. Nat Rev Drug Discov (2012) 11:763–76. 10.1038/nrd3794 23023676

[56] Lubberts E. The IL-23-IL-17 axis in inflammatory arthritis. Nat Rev Rheumatol (2015) 11:562. 10.1038/nrrheum.2015.128 26369609

[57] Kurschus FC Croxford AL Heinen AP Wortge S Ielo D Waisman A. Genetic proof for the transient nature of the Th17 phenotype. Eur J Immunol (2010) 40:3336–46. 10.1002/eji.201040755 21110317

[58] Geboes L Dumoutier L Kelchtermans H Schurgers E Mitera T Renauld JC Proinflammatory role of the Th17 cytokine interleukin-22 in collagen-induced arthritis in C57BL/6 mice. Arthritis Rheum (2009) 60:390–5. 10.1002/art.24220 19180498

[59] Marijnissen RJ Koenders MI Smeets RL Stappers MH Nickerson-Nutter C Joosten LA Increased expression of interleukin-22 by synovial Th17 cells during late stages of murine experimental arthritis is controlled by interleukin-1 and enhances bone degradation. Arthritis Rheum (2011) 63:2939–48. 10.1002/art.30469 21618207

[60] Pineda MA Rodgers DT Al-Riyami L Harnett W Harnett MM. ES-62 protects against collagen-induced arthritis by resetting interleukin-22 toward resolution of inflammation in the joints. Arthritis Rheumatol (2014) 66:1492–503. 10.1002/art.38392 24497523 PMC4737104

[61] da Rocha LF Jr Duarte AL Dantas AT Mariz HA Pitta Ida R Galdino SL Increased serum interleukin 22 in patients with rheumatoid arthritis and correlation with disease activity. J Rheumatol (2012) 39:1320–5. 10.3899/jrheum.111027 22589261

[62] Zhao L Jiang Z Jiang Y Ma N Zhang Y Feng L IL-22+ CD4+ T cells in patients with rheumatoid arthritis. Int J Rheum Dis (2013) 16:518–26. 10.1111/1756-185X.12099 24164838

[63] Kim KW Kim HR Park JY Park JS Oh HJ Woo YJ Interleukin-22 promotes osteoclastogenesis in rheumatoid arthritis through induction of RANKL in human synovial fibroblasts. Arthritis Rheum (2012) 64:1015–23. 10.1002/art.33446 22034096

[64] Smolen JS Landewe R Bijlsma J Burmester G Chatzidionysiou K Dougados M EULAR recommendations for the management of rheumatoid arthritis with synthetic and biological disease-modifying antirheumatic drugs: 2016 update. Ann Rheum Dis (2017) 76:960–77. 10.1136/annrheumdis-2016-210715 28264816

